# Identification of Candidate Protein Markers in Skeletal Muscle of Laminin-211-Deficient CMD Type 1A-Patients

**DOI:** 10.3389/fneur.2019.00470

**Published:** 2019-05-07

**Authors:** Heike Kölbel, Denisa Hathazi, Matthew Jennings, Rita Horvath, Andreas Roos, Ulrike Schara

**Affiliations:** ^1^Department of Pediatric Neurology, Developmental Neurology and Social Pediatrics, University of Duisburg-Essen, Essen, Germany; ^2^Leibniz-Institut für Analytische Wissenschaften -ISAS- e.V., Dortmund, Germany; ^3^Department of Clinical Neuroscience, University of Cambridge, Cambridge, United Kingdom

**Keywords:** laminin-211, laminin-α2, congenital muscular dystrophy, agrin, NudC domain-containing protein 2, muscle proteomics

## Abstract

Laminin-211 deficiency leads to the most common form of congenital muscular dystrophy in childhood, MDC1A. The clinical picture is characterized by severe muscle weakness, brain abnormalities and delayed motor milestones defining MDC1A as one of the most severe forms of congenital muscular diseases. Although the molecular genetic basis of this neurological disease is well-known and molecular studies of mouse muscle and human cultured muscle cells allowed first insights into the underlying pathophysiology, the definition of marker proteins in human vulnerable tissue such as skeletal muscle is still lacking. To systematically address this need, we analyzed the proteomic signature of laminin-211-deficient vastus muscle derived from four patients and identified 86 proteins (35 were increased and 51 decreased) as skeletal muscle markers and verified paradigmatic findings in a total of two further MDC1A muscle biopsies. Functions of proteins suggests fibrosis but also hints at altered synaptic transmission and accords with central nervous system alterations as part of the clinical spectrum of MDC1A. In addition, a profound mitochondrial vulnerability of the laminin-211-deficient muscle is indicated and also altered abundances of other proteins support the concept that metabolic alterations could be novel mechanisms that underline MDC1A and might constitute therapeutic targets. Intersection of our data with the proteomic signature of murine laminin-211-deficient gastrocnemius and diaphragm allowed the definition of nine common vulnerable proteins representing potential tissue markers.

## Introduction

Congenital muscular dystrophies (CMD) comprise a heterogenous group of genetically caused neuromuscular diseases with muscle weakness apparent at birth or in the first 6 months of life. Most of the different subtypes are of autosomal recessive inheritance (1). Laminin-211 (formerly merosin) -deficient CMD type 1A (MDC1A) is caused by recessive mutations in the *LAMA2* gene (encoding for the α2 subunit of laminin-211) and constitutes approximately 10–30% of total CMD cases in the European population. Laminin-211 is expressed in the brain vasculature, the skeletal muscle basal lamina as well as in the myotendinous and neuromuscular junctions (2). White matter T2 signal hyperintensity as reflection of increased interstitial water content occurs in almost all patients after 6 months of age (3, 4). MDC1A-patients suffer from muscular weakness associated with elevated serum creatine kinase (CK) levels, poor suck and cry, multiple joint contractures and delayed motor development. Most of the MDC1A-patients never achieve independent ambulation (5–7). Extramuscular manifestations include seizures in 30% of patients, demyelinating neuropathy and CNS abnormalities such as polymicrogyria and cortical bandlike heterotopia. Mental retardation rarely occurs (8). Whereas patients with a complete deficiency of laminin-211 present with a severe clinical spectrum of the disease, a partial deficiency of the protein leads to milder phenotypes (6). In 2011, Gawlik and Durbeej speculated that the pathogenicity of *LAMA2* mutations, which disrupt the assembly of the corresponding laminin-211 protein with other basal lamina components, explains the full penetrance of the phenotype (9).

Laminin is a cell-adhesion molecule localized to the basement membrane of skeletal muscle. The biological functions of laminins such as modulation of cytoskeleton and intracellular signaling pathways are accomplished via the interaction with transmembrane receptors which—in skeletal muscle—are represented by dystroglycan and integrin α7β1 as the two major receptors for laminin-211 (10, 11). In this context, laminin has also been postulated to protect the muscle fibers from damage under the constant stress of contractions (9). Notably, while laminin-211 (composed of α2, β1, and γ1 chains) was first isolated from placenta and originally called merosin (12), it is now well established that laminin-211 is the main laminin isoform in skeletal muscle (12–14). In this context, it is important to note that laminin-211 function has been linked to muscle development and through the formation of laminin networks also to cytoskeleton and intracellular signaling pathways. Moreover, it is believed that laminin-211 influences signal transmission events and muscle innervation via modulation of NMJ-integrity and function (9).

Although clinical features of *LAMA2*-patients, especially regarding the manifestation in skeletal muscle are well described, molecular signatures of muscle pathology defining protein markers of the effect of loss of functional laminin-211 remain elusive. Utilizing label free mass spectrometry-based protein quantification, we here systematically address laminin-211-deficiency-related protein changes to define tissue biomarkers in skeletal muscle of MDC1A-patients from the severe disease spectrum. This in turn can provide potential insights into the underlying pathophysiology and thus represent valuable outcome measures for therapeutic intervention concepts such as the emerging gene therapy (15). Hence, data presented in this article provides important molecular insights into MDC1A-related pathophysiology due to alterations in protein composition beyond the extra-cellular matrix, an important aspect for the definition of further or alternative therapeutic intervention concepts.

## Materials and Methods

The muscle biopsy specimen investigated in this study have been initially collected for diagnostic purposes. Written informed consent was obtained from the participants (or rather their legal guardians) for the participation into the subsequent research as well as for publication of the findings (including any potentially-identifying information). Ethical approval was not required as per the local legislation.

### Immunofluorescence-Based Studies in Muscle Biopsies

Five micrometer cryosections of muscle biopsy specimen were generated, dried and afterwards re-hydrated in phosphate-buffered saline (PBS) followed by exposure to primary antibodies (Table 1) diluted in 1% bovine serum albumin (BSA). After incubation of the sections in a wet-chamber for one hour at 25°C (incubator), those were washed three times in PBS. Next, the secondary antibodies (Alexa 488-conjugated) 1:500 diluted in 1% BSA were added and sections were again incubated in a wet-chamber for one hour at 25°C. Prior sections were finally covered in mounting medium (Dako), three washing steps in PBS were performed.

**Table 1:** List of antibodies used for the immunofluorescence studies.

**Primary antibody**	**Company**	**Number**	**Dilution**	**Secondary antibody**
GPI	Abcam	66340	1:200	Mouse anti-goat 488
Lama α5	Millipore	MAB 1924	1:500	Mouse anti-goat 488
Glypican-1	Abcam	55971	1:100	Donkey anti-rabbit 488
Agrin	Millipore	MAB 5204	1:20	Mouse anti-goat 488
Spectrin	Leica	SPEC 1-CE	1:100	Mouse anti-goat 594
Lama α2C	Millipore	MAB1922	1:500	Mouse anti-goat 488
Lama α2N	Leica	MEROSIN-CE	1:50	Mouse anti-goat 488
Mouse anti goat IgG 488		Alexa A-11029	1:500	
Mouse anti goat IgG 594		Alexa A-11032	1:500	
Donkey anti rabbit IgG 594		Alexa A-2106	1:500	

### Immunoblot-Based Studies on Whole Muscle Protein Extracts

Twenty micrograms of patient skeletal muscle protein lysates were prepared using NuPAGE LDS sample buffer (Thermo) and NuPAGE reducing agent, denatured at room temperature for 10 minutes and loaded into 4–12% gradient SDS-PAGE gels (Thermo, NP0322). Proteins were separated and transferred to a PVDF membrane using an iBlot2 dry transfer system (Thermo). PVDF membranes were blocked using 5% milk in Tris buffer-saline with 1% tween-20 (TBS-T) at room temperature for 1 hour before probing using monoclonal antibodies targeting NDUFB8, SDHB, UQCRC2, COX2, and ATP5A (Abcam, ab110411) as well as antibodies targeting GAPDH (Abcam, ab8245) and VDAC1 (Abcam, ab14734). Membranes were washed in TBS-T and probed again for 1 h with HRP-conjugated anti-Mouse IgG (Abcam, ab97023) prior to chemiluminescent imaging. Quantification was performed using ImageJ.

### Proteomic Profiling in Human Skeletal Muscle

Ammonium hydrogen carbonate (NH_4_HCO_3_), anhydrous magnesium chloride (MgCl_2_), guanidine hydrochloride (GuHCl), iodoacetamide (IAA), and urea were purchased from Sigma-Aldrich, Steinheim, Germany. Tris base was obtained from Applichem Biochemica, Darmstadt, Germany and Sodium dodecyl sulfate (SDS) was purchased from Carl Roth, Karlsruhe, Germany. Dithiothreitol (DTT), EDTA-free protease inhibitor (Complete Mini) tablets were obtained from Roche Diagnostics, Mannheim, Germany. Sodium chloride (NaCl) and calcium chloride (CaCl_2_) were from Merck, Darmstadt. Sequencing grade modified trypsin was from Promega, Madison, WI USA. Benzonase® Nuclease was purchased from Novagen. Bicinchoninic acid assay (BCA) kit was acquired from Thermo Fisher Scientific, Dreieich, Germany. All chemicals for ultra-pure HPLC solvents such as formic acid (FA), trifluoroacetic acid (TFA) and acetonitrile (ACN) were obtained from Biosolve, Valkenswaard, The Netherlands.

#### Cell Lysis, Sample Preparation and Trypsin Digestion

In total eight samples derived from four healthy controls (gender- and age-matched) and four MDC1A-patients were processes independently. All muscle samples were collected from the mid portion of vastus lateralis. Approximately 10 slices of 10 μm of muscle were lysed in 50 μL of lysis buffer (50 mM Tris-HCl (pH 7.8) 150 mM NaCl, 1% SDS, and Complete Mini) using a manual glass grinder. Then samples were centrifuged for 5 min at 4°C and 5.000 g. Protein concentration of the supernatant was determined by BCA assay (according to the manufacturer's protocol). In order to reduce the cysteines 10 mM of DTT were added to the samples followed by incubation at 56°C for 30 min. Next, the free thiol groups were alkylated with 30 mM IAA at room temperature (RT) in the dark for 30.

Sample digestion and cleanup were performed using filter-aided sample preparation (FASP) as described previously (16, 17) with some minor changes. Briefly, 100 μg of protein lysate was diluted 10-fold with freshly prepared 8 M urea/100 mM Tris-HCl (pH 8.5) buffer (18) and placed on a Microcon centrifugal device (30 KDa cutoff). Afterwards, the filter was centrifuged at 13,500 g at RT for 15 min (all the following centrifugation steps were performed under the same conditions). Three washing steps were carried out with 100 μL of 8 M urea/100 mM Tris-HCl (pH 8.5). For buffer exchange, the device was washed thrice with 100 μL of 50 mM NH_4_HCO_3_ (pH 7.8). Next, 100 μL of the digestion buffer (trypsin (Promega), 1:25 w/w, protease to substrate, (0.2 M GuHCl and 2 mM CaCl_2_ in 50 mM NH_4_HCO_3_ pH 7.8), was added to the filter which contains the bound proteins and the samples were incubated at 37°C for 14 h. Resulting tryptic peptides were recovered by centrifugation with 50 μL of 50 mM NH_4_HCO_3_ followed by 50 μL of ultra-pure water. In the final step, tryptic peptides were acidified by adding 5 μl of 10 % TFA (v/v). The digests were quality controlled as described previously (19).

#### LC-MS/MS Analysis

Samples (technical duplicates) were measured using an Ultimate 3000 nano RSLC system coupled to an Orbitrap Fusion Lumos mass spectrometer (both Thermo Scientific) and analyzed in a randomized order to minimize systematic errors. Firstly, peptides were preconcentrated on a 100 μm × 2 cm C18 trapping column for 10 min using 0.1 % TFA (v/v) at a flow rate of 20 μL/min. Next the separation of the peptides was performed on a 75 μm × 50 cm C18 main column (both Pepmap, Thermo Scientific) with a 120 min LC gradient ranging from 3 to 35 % of 84 % ACN, 0.1 % FA (v/v) at a flow rate of 230 nL/min. MS^1^ spectra was acquired in the Orbitrap from 300 to 1,500 m/z at a resolution of 120,000 using the polysiloxane ion at *m/z* 445.12003 as lock mass (20), with maximum injection times of 50 ms. Next, top 10 most intense signals were selected for fragmentation by HCD with a collision energy of 30%. MS^2^ spectra were acquired in the ion trap at a resolution of 120,000, with maximum injection times of 300 ms and a dynamic exclusion of 15 s. The ACG target was set at 2.0 × 10^3^ ions for MS^2^ and 2.0 × 10^5^ for MS^2^.

#### Label Free Data Analysis

Data analysis of the acquired label free quantitative MS data was performed using the Progenesis LC-MS software from Non-linear Dynamics (Newcastle upon Tyne, U.K.). Alignment of MS raw data was conducted by Progenesis, which automatically selected one of the LC-MS files as reference. Next, peak picking was performed and only features within retention time and *m/z* windows from 0 to 120 min and 300–1,500 m/z, with charge states +2, +3, and +4 were considered for peptide statistics, analysis of variance (ANOVA). The MS/MS spectra were exported as peak lists which were searched against a concatenated target/decoy version of the mouse Uniprot database (Homo sapiens with 20273 entries, downloaded 22.07.2017) using Mascot 2.4 (Matrix Science), MS-GF+, and X!Tandem Jackhammer (2013.06.15) with the help of searchGUI 1.14.4 (21). Trypsin with a maximum of two missed cleavages was selected as enzyme. Carbamidomethylation of Cys was set as fixed and oxidation of Met was selected as variable modification. MS and MS/MS tolerances were set to 10 ppm and 0.5 Da, respectively. Combined search results were filtered at a false discovery rate (FDR) of 1 % on the protein level and exported using PeptideShaker 0.28.0 (http://code.google.com/p/peptide-shaker/). Data was reimported into Progenesis and peptide sequences containing oxidized Met were excluded for further analysis. Only proteins that were quantified with unique peptides were exported. For each protein, the average of the normalized abundances (obtained from Progenesis) was calculated and used to determine the ratios between patients and control. Only proteins which were (*i*) commonly quantified in all the replicates with (*ii*) one unique peptides, (*iii*) an ANOVA *p*-value of ≤0.05 (Progenesis) and (*iv*) an average ratio ≤ log_2_ 0.99or ≥ log_2_−0.9 were considered as up respectively down regulated.

#### Data Plotting and Pathway Analysis

All data was plotted using Origin 6.0 and Adobe Illustrator. For pathway analysis, the GO ontology, KEGG and Reactome were used and data manually filtered for relevant pathways. The Proteomap was the online tool available (https://www.proteomaps.net/). The annotation of these proteomaps is based on the KEGG database platform, each protein is shown by a polygon, and functionally relevant proteins are arranged as neighbors. Additionally, polygon areas represent protein abundances weighted by protein size.

## Results

### Clinical and Genetic Findings

We followed six patients diagnosed with CMD type 1A in the Department of Neuropediatrics of the University Children's Hospital, University Duisburg-Essen, in a tertiary care setting. Mutations in *LAMA2* were found in all patients; however, one patient, who was severely affected, was lost of follow-up at the age of 2 years (patient 1).

Table 2 summarizes the molecular genetic, clinical and neuroradiologic findings. All six patients presented with symptoms within the first 4 months of life: poor head control, generalized muscular hypotonia and muscular weakness, poor spontaneous movements, delayed motor milestones, and high levels of creatine kinase (CK). The highest measured CK levels are listed in Table 2. The six children never achieved ambulation. Patient 5 was able to walk with support, but after a febrile virus infection with rhabdomyolysis at the age of 20 months she had a dramatic loss of motor function. Currently, at the age of 35 months, she showed a recovery with the ability to stand with support. All patients had dysphagia, recurrent chest infections and need varying levels of pulmonal support (i.e., cough assist). Additionally, joint contractures were present in all six patients. In patients 2–6, the underlying genetic mutations segregate with the phenotypes.

**Table 2:** Clinical findings in MDC1A patients included in the overall study.

**Patient**	**Age at last visit**	**Mutation**	**Achieved motor milestone**	**Onset of symptoms**	**Age at biopsy**	**Inheritance**	**CK**	**Cardial function**	**Pulmonal function**	**cMRI**
1 (female)	2 y		Free sitting after being placed at 14 months of age	Reduced child movements during pregnancy. pp: muscular weakness	2 m	Unknown	4,000	Normal	Normal	At 4m of age normal
2 (male)	7 y	Homozygous: c.5134_5153del (leading to a premature stop-codon; p.Arg1712Glufs*4)	Free sitting at 18 months of age, able to scoot on his bottom	At the 3 months of age not able to lift the head and poor head control	9 m	Recessive	3,434	Normal	Normal	Not done
3 (male)	6 y	Homozygous: c.7898+2T>G (presumably leading to skipping of exon 56; pathogenicity of an adjacent mutation) (c.7898+1G>T) has already been reported (22)	Free sitting at 10 months of age after being placed	pp: muscular weakness	14 m	Recessive	1,584	Corrected QT interval of 451 m/sec (< 440)ECHO normal	Normal	Leuko-encephalo-pathy
4 (male)	4 y	Compound heterozygous: c.397_397delG & c.1884+2T>C	Free sitting at 30 months of age after being placed sitting after being placed	pp: muscular weakness	4 m	Recessive	7,400	Normal	Normal	Leuko-encephalo-pathy
5 (female)	3y	Homozygous: c.8244+1G>A(rs749522728); (23)	Walking at 18 months of age with support	At the 4 months of age not able to lift the head and muscular weakness. Rhabdomyolysis at the age of 20 months	26m	Recessive	2,7000	Normal	Normal	Not done
6 (female)	2y	Homozygous: deletion of exons 31-40 (diagnosed via MLPA)	Free sitting at 14 months of age after being placed sitting after being placed	At the 3 months of age not able to lift the head and muscular weakness.	14m	Recessive	5,200	Normal	Normal	Leuko-encephalo-pathy

*Y, years; m, month; pp, post-partal; CK, creatine kinase; cMRI, cranial magnetic resonance imaging*.

**Figure 1:**
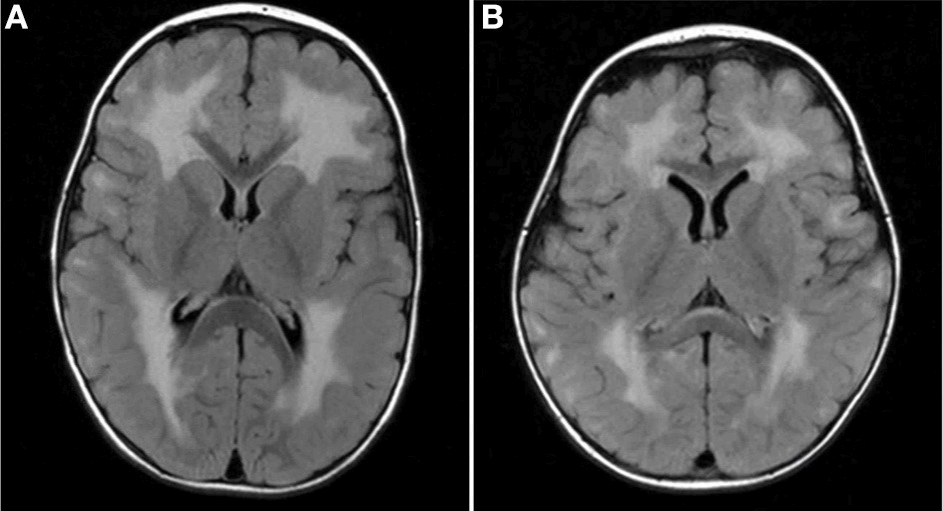
MRI findings in MDC1A-patients included in the study. Brain MRI TIRM (Turbo-Inversion Recovery-Magnitude, axial) show diffuse symmetrical white matter lesions predominantly in the periventricular regions and no involvement of thalamus or brainstem in both cases. **(A)** Patient 3 with expanded supratentorial leukencephalopathy at two years of age. **(B)** Patient 4 diffuse leukencephalopathy.

Four of our six patients underwent brain MRI, and three of the four showed white matter lesions (WMLs) on T2-weighted brain MRI images. The WMLs were characterized by a diffuse symmetrical distribution in cerebral areas that are normally myelinated in the developing brain (Figure 1) without involvement of thalamus or brainstem in both cases. Lissencephaly could not be detected in our patients. Patient 1 had a brain MRI performed at the age 4 months, which might be too early to detect WML in laminin-211-deficiency. All patients had normal cognitive development with no seizures being reported.

### Muscle Biopsy Findings

**Figure 2:**
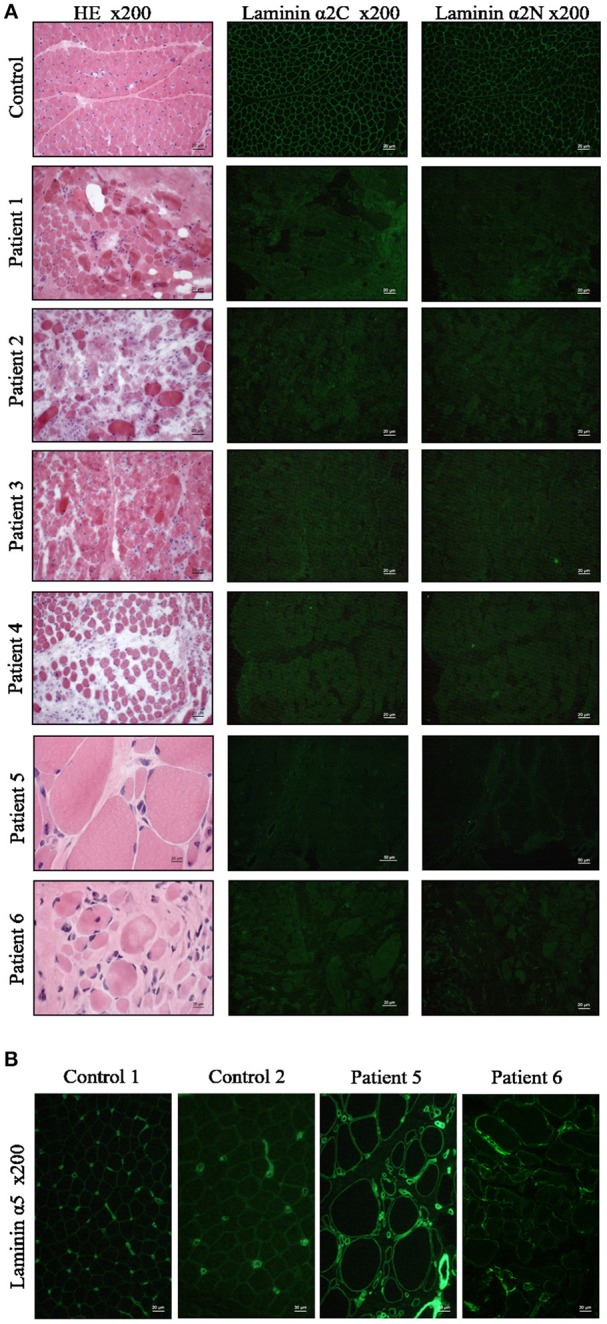
Muscle biopsy findings in MDC1A-patients included in the study. **(A)** Muscle biopsy cryostat sections stained with hematoxylin and eosin (HE) and immunofluorescence staining with anti-laminin-211 antibodies reveals absent immunoreactivity in the patient-derived sections. **(B)** Immunofluorescence staining of muscle cryostat sections with anti-laminin-511 antibody reveals in increased immunoreactivity in muscle biopsies of MDC1A-patients as indicated for two representative cases shown here. Ctrl, control muscle; numbers 1, 2, 3, 4, 5, and 6 refer to muscle biopsies derived from MDC1A-patients with the corresponding patient numbers listed in Table 2.

Frozen biopsied muscle tissue samples from all six patients were analyzed and showed severe features of muscular dystrophy including increased extracellular connective tissue with fibrosis, cell necrosis, numerous central nuclei, and high fiber size variability (Figure 2A). All patients presented total absence of laminin-211 in our immunofluorescence-based microscopic examinations (Figure 2A). Moreover, laminin-511 was mild do moderately increased in the muscle biopsy specimen of the six patients as shown for two representative cases in Figure 2B. As controls, we selected subjects who had underwent a muscle biopsy, but who were found not to have any signs of a skeletal muscle disease.

### Loss of LAMA2 Induces Changes in General Protein Composition in Human Skeletal Muscle

**Figure 3:**
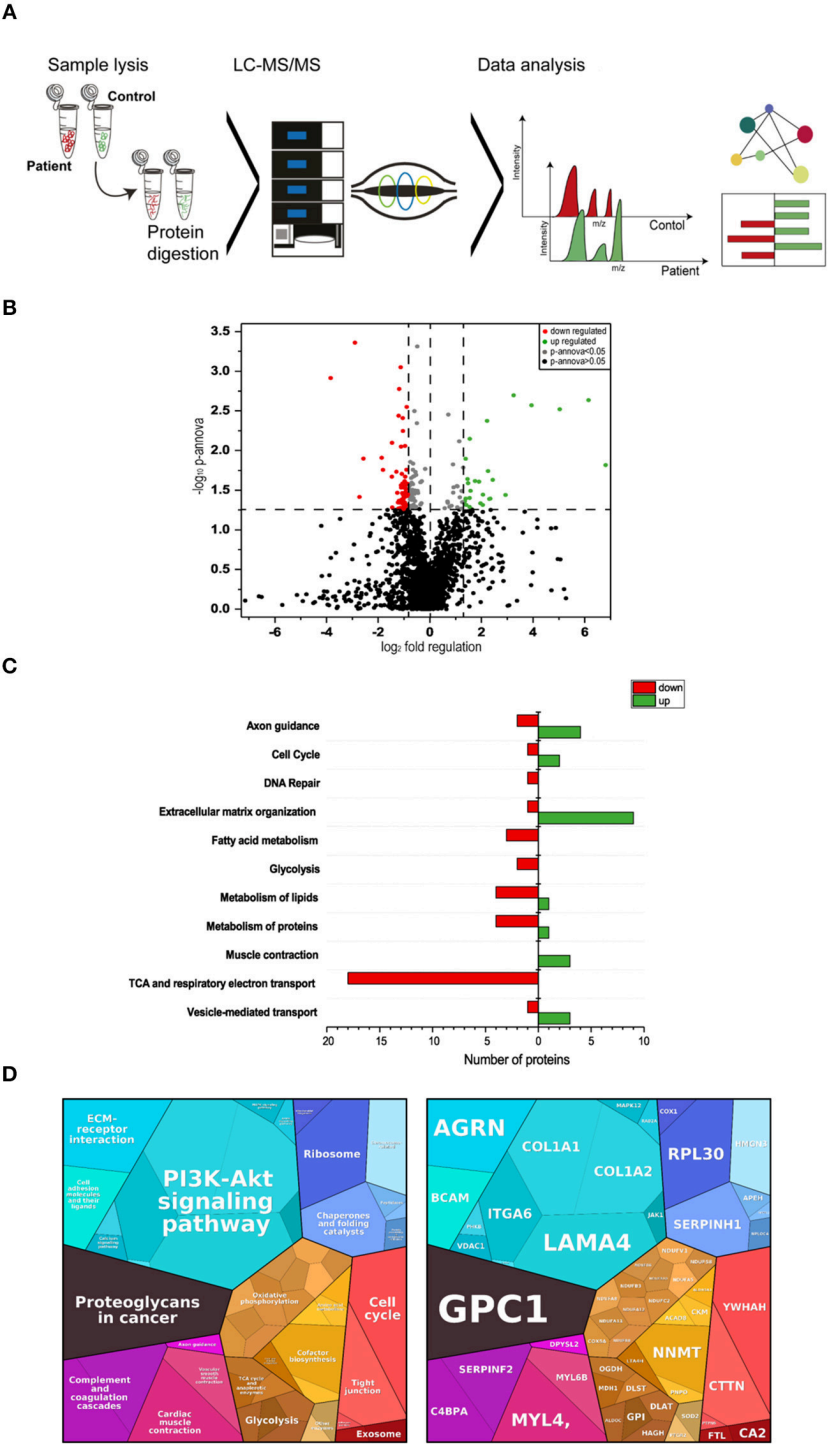
Proteomic profiling of four MDC1A-patient derived muscles. **(A)** Methodological workflow applied in the study. **(B)** Results of our label-free proteomic profiling are shown as a volcano plot. All points (each one represents a protein) over the horizontal line have a statistically significant p-ANOVA of ≤0.05. In total, 51 proteins are decreased (red points) and 35 proteins are increased in abundance (green points) in laminin-211-deficient skeletal muscle. **(C)** Results of an *in silico* pathway analysis of proteins altered in abundance utilizing DAVID, KEGG, and Reactome platforms indicating alterations of ECM protein composition, cellular metabolism, axon guidance, cell cycle, and DNA repair, muscle contraction and of vesicular protein transport. **(D)** Results of an *in silico* pathway analysis of proteins altered in abundance utilizing Proteomap platform confirming the perturbed cellular functions indicated by the results of the previous pathway analysis and moreover indicating perturbed function of cellular signaling cascades such as PI3K-Akt signaling.

Proteomic profiling is a useful approach to obtain unbiased insights into the molecular etiology of diseases such as muscular disorders (24, 25). To identify, molecular marker proteins for muscle fiber vulnerability due to the loss of functional laminin-211 (protein encoded by *LAMA2*), the proteomic signature of muscle biopsy specimen of four MDC1A-patients (patients 1–4; see Table 2) from the severe spectrum of the disease was compared to the signature in four controls. Using liquid chromatography coupled to tandem mass spectrometry (LC-MS/MS) (label free protein quantification) (Figure 3), we quantified 1977 proteins out of which 86 proteins presented (4.3%) with statistically significant altered abundances (35 were increased and 51 decreased; Table 3). The considerable decrease of laminin-211 (−3.84; log_2_) in the patient-derived samples hereby reflects the sensitivity of our proteomic profiling approach as well as the reliability of the data. To obtain insights into the molecular etiopathology of MDC1A, pathway analyses were performed utilizing DAVID (https://david.ncifcrf.gov/), KEGG (www.genome.jp/kegg/pathway.html), Reactome (www.reactome.org) and Proteomap (www.proteomaps.net). In addition, information concerning subcellular localization and function has been extracted from uniport (www.uniprot.org) for each protein altered in abundance to provide a complete picture of the molecular basis of MDC1A manifestation in skeletal muscle (Table 3). Data analysis via diverse pathway analysis tools revealed potential changes in axon guidance, cell cycle and DNA-repair, extracellular matrix organization, metabolism of fatty acids, sugar, lipids and proteins. In addition, protein changes seem to impact on vesicular transport machinery, TCA and respiratory electron transport as well as proper muscle contraction (Figure 3C). Data analysis via Proteomap confirmed the impact of detected protein changes on cell cycle, cellular metabolism, muscle contraction as well as on the composition of the extracellular matrix. Based on the functional properties of some of the proteoglycans with altered abundances such as COL1A1 and -A2, LAMA4 and ITGA6, changes in PI3K/Akt-signaling as a pathway involved in a variety of cellular functions has been highlighted by the Proteomaps-representation (Figure 3D). This molecular observation accords with a postulated function of laminin-211 in signal transmission (9).

**Table 3:** List of proteomic findings.

**Accession**	**Pep. count/ uniq. Pep**.	**Anova (p)**	**Description**	**Localization**	**Fold of regu-lation**	**Log2**	**Function**
P15502	1/1	0.02	Elastin (*ELN*)	ECM	112.37	6.81	Extracellular matrix structural constituent
Q9GZT8	2/1	0.00	NIF3-like protein 1 (*NIF3L1*)	Nucleus	70.76	6.14	Negatively regulating the expression of genes involved in neuronal differentiation
P09486	2/2	0.00	SPARC (*SPARC*)	ECM	32.77	5.03	Regulates cell growth through interactions with the extracellular matrix and cytokines
P20472	7/5	0.00	Parvalbumin alpha (*PVALB*)	Nucleus	15.34	3.94	Involved in muscle-relaxation after contraction
P35052	1/1	0.00	Glypican-1 (*GPC1*)	ECM	9.51	3.25	Binds collagen and participates in Schwann cell myelination
Q8N414	1/1	0.04	PiggyBac transposable element-derived protein 5 (*PGBD5*)	Nucleus	7.63	2.93	Targets regulatory elements and tumor suppressor genes and promotes cell transformation
Q16363	18/17	0.02	Laminin subunit alpha-4 (*LAMA4*)	ECM	5.42	2.44	Mediates attachment, migration and organization of cells into tissues during embryonic development by interacting with other extracellular matrix components
P02452	34/31	0.04	Collagen alpha-1(I) chain (*COL1A1*)	ECM	5.06	2.34	Extracellular matrix structural constituent
P08123	15/15	0.04	Collagen alpha-2(I) chain (*COL1A2*)	ECM	4.93	2.30	Extracellular matrix structural constituent
O00468	5/5	0.02	Agrin (*AGRN*)	Synaptic cleft	4.77	2.25	Formation and the maintenance of the NMJ and postsynaptic differentiation
Q8WVJ2	2/2	0.00	NudC domain-containing protein 2 (*NUDCD2*)	Cyto-skeleton	4.66	2.22	Regulates the LIS1/dynein pathway by stabilizing LIS1 with Hsp90 chaperone
Q04917	10/5	0.04	14-3-3 protein eta (*YWHAH*)	Cytosol	4.17	2.06	Promotes myosin II turnover in the cell cortex and modulates cortical tension, cell shape, and cytokinesis (doi: 10.1074/jbc.M117.819391)
P62888	4/4	0.05	60S ribosomal protein L30 (*RPL30*)	Cytosol	4.14	2.05	Protein synthesis
P29400	2/1	0.05	Collagen alpha-5(IV) chain (*COL4A5*)	ECM	3.90	1.96	Extracellular matrix structural constituent
P12829	14/11	0.02	Myosin light chain 4 (*MYL4*)	Cyto-skeleton	3.75	1.91	Regulatory light chain of myosin
Q14314	2/2	0.02	Fibroleukin (*FGL2*)	ECM	3.38	1.75	Playa a role in physiologic lymphocyte functions
P02766	9/8	0.03	Transthyretin (TTR)	ECM	2.98	1.58	Protects against misfolding and the formation of amyloid fibrils
Q14247	2/2	0.01	Src substrate cortactin (SRC8_HUMAN)	Plasma membrane	2.92	1.55	Plays a role in the regulation of neuron morphology, axon growth and formation of neuronal growth cones
P08697	5/5	0.05	Alpha-2-antiplasmin (*SERPINF2*)	ECM	2.91	1.54	Serine protease inhibitor
P23229	2/2	0.04	Integrin alpha-6 (*ITGA6*)	Plasma membrane	2.90	1.54	Integrin alpha-6/beta-1 is a receptor for laminin
P50454	14/14	0.03	Serpin H1 (*SERPINH1*)	Endoplas-mic Reticulum	2.80	1.48	Chaperone in the biosynthetic pathway of collagen
Q9BZQ8	5/5	0.02	Protein Niban (*FAM129A*)	Endoplas-mic Reticulum	2.75	1.46	Involved in the Endoplasmic Reticulum stress response
Q15417	5/5	0.05	Calponin-3 (*CNN3*)	Cyto-skeleton	2.66	1.41	Thin filament-associated protein that is implicated in the regulation and modulation of muscle contraction
P04003	12/10	0.04	C4b-binding protein alpha chain (*C4BPA*)	ECM	2.62	1.39	Controls the classical pathway of complement activation
P50895	10/10	0.01	Basal cell adhesion molecule (*BCAM*)	Plasma membrane	2.62	1.39	Laminin alpha-5 receptor
P40261	8/8	0.04	Nicotinamide N-methyltransferase (*NNMT*)	Cytoplasm	2.54	1.35	Catalyzes the N-methylation of nicotinamide and other pyridines to form pyridinium ions
Q15651	3/1	0.02	High mobility group nucleosome-binding domain-containing protein 3 (*HMGN3*)	Nucleus	2.45	1.29	Regulates chromatin-dependent processes such as transcription, DNA-replication and -repair
Q8N3D4	3/3	0.04	EH domain-binding protein 1-like protein 1 (*EHBP1L1*)	Endosome	2.39	1.26	Rab effector protein playing a role in vesicle trafficking
Q9UEY8	3/3	0.03	Gamma-adducin (*ADD3*)	Plasma membrane	2.35	1.23	Membrane-cytoskeleton-associated protein that promotes the assembly of the spectrin-actin network and plays a role in actin filament capping
Q8NC56	1/1	0.05	LEM domain-containing protein 2 (*LEMD2*)	Nucleus	2.34	1.22	Involved in nuclear structure organization
P02760	10/10	0.04	Protein AMBP (*AMBP*)	ECM	2.24	1.16	Elastase inhibitor
Q96AQ6	19/18	0.01	Pre-B-cell leukemia transcription factor-interacting protein 1 (*PBXIP1*)	Nucleus	2.20	1.14	Tethers estrogen receptor-alpha (ESR1) to microtubules and allows them to influence estrogen receptors-alpha signaling
Q14BN4	5/5	0.03	Sarcolemmal membrane-associated protein (*SLMAP*)	Plasma membrane	2.10	1.07	Plays a role during myoblast fusion
O43488	4/4	0.05	Aflatoxin B1 aldehyde reductase member 2 (*AKR7A2*)	Golgi apparatus	2.09	1.07	Plays an important role in producing the neuromodulator gamma-hydroxybutyrate (GHB)
P14649	25/22	0.03	Myosin light chain 6B (*MYL6B*)	Cytoskeleton	2.08	1.05	Regulatory light chain of myosin
P09960	11/10	0.03	Leukotriene A-4 hydrolase (*LTA4H*)	Cytoplasm	0.53	−0.92	Biosynthesis of proinflammatory mediator leukotriene B4
Q16775	6/6	0.04	Hydroxyacylglutathione hydrolase, mitochondrial (*HAGH*)	Mito-chondria	0.53	−0.92	Catalyzes the hydrolysis of S-D-lactoyl-glutathione to form glutathione and D-lactic acid
O43598	2/2	0.03	2′-deoxynucleoside 5′-phosphate N-hydrolase 1 (*DNPH1*)	Nucleus	0.53	−0.93	Catalyzes the cleavage of the N-glycosidic bond of deoxyribonucleoside 5′-monophosphates
Q3L8U1	1/1	0.05	Chromodomain-helicase-DNA-binding protein 9 (*CHD9*)	Nucleus	0.52	−0.95	Transcriptional coactivator for PPARA and possibly other nuclear receptors
Q9H0R4	1/1	0.03	Haloacid dehalogenase-like hydrolase domain-containing protein 2 (*HDHD2*)	ECM	0.52	−0.95	Phosphotase activity
P06744	24/24	0.02	Glucose-6-phosphate isomerase (*GPI*)	ECM	0.52	−0.95	Neurotrophic factor for spinal and sensory neurons
P10515	11/11	0.03	Dihydrolipoyllysine-residue acetyltransferase component of pyruvate dehydrogenase complex (*DLAT*)	Mito-chondria	0.51	−0.96	Catalyzes the overall conversion of pyruvate to acetyl-CoA and CO_2_
P30086	21/20	0.01	Phosphatidylethanolamine-binding protein 1 (*PEBP1*)	Cytoplasm	0.51	−0.96	Involved in the function of the presynaptic cholinergic neurons of the central nervous system; increases the production of choline acetyltransferase but not acetylcholinesterase
Q9UI09	3/2	0.05	NADH dehydrogenase [ubiquinone] 1 alpha subcomplex subunit 12 (*NDUFA12*)	Mito-chondria	0.51	−0.96	Accessory subunit of the mitochondrial membrane respiratory chain NADH dehydrogenase (complex I)
Q02218	29/26	0.04	2-oxoglutarate dehydrogenase, mitochondrial (*OGDH*)	Mito-chondria	0.51	−0.96	2-oxoglutarate dehydrogenase (E1) component of the 2-oxoglutarate dehydrogenase complex
P09972	11/2	0.02	Fructose-bisphosphate aldolase C (*ALDOC*)	Cytoplasm	0.51	−0.97	Glycolysis
P49821	18/16	0.03	NADH dehydrogenase [ubiquinone] flavoprotein 1 (*NDUFV1*)	Mito-chondria	0.50	−0.99	Core subunit of the mitochondrial membrane respiratory chain NADH dehydrogenase (Complex I)
O95298	2/2	0.05	NADH dehydrogenase [ubiquinone] 1 subunit C2 (*NDUFC2*)	Mito-chondria	0.50	−0.99	Accessory subunit of the mitochondrial membrane respiratory chain NADH dehydrogenase (Complex I)
P21796	22/20	0.05	Voltage-dependent anion-selective channel protein 1 (*VDAC1*)	Mito-chondria	0.50	−0.99	Forms a channel through the mitochondrial outer membrane; allows diffusion of small hydrophilic molecules
P00918	15/12	0.05	Carbonic anhydrase 2 (*CA2*)	Plasma membrane	0.50	−0.99	Contributes to intracellular pH regulation
Q16718	6/6	0.03	NADH dehydrogenase [ubiquinone] 1 alpha subcomplex subunit 5 (*NDUFA5*)	Mito-chondria	0.49	−1.00	Accessory subunit of the mitochondrial membrane respiratory chain NADH dehydrogenase (Complex I)
P83111	6/6	0.04	Serine beta-lactamase-like protein LACTB, (*LACTB*)	Mito-chondria	0.49	−1.01	Serine protease that acts as a regulator of mitochondrial lipid metabolism
Q8TCA0	6/6	0.05	Leucine-rich repeat-containing protein 20 (*LRRC20*)	Information not available	0.49	−1.01	Information not available
P20674	8/8	0.04	Cytochrome c oxidase subunit 5A, (*COX5A*)	Mito-chondria	0.49	−1.02	This is the heme A-containing chain of cytochrome c oxidase
Q96A26	4/4	0.04	Protein FAM162A (*F162A*)	Mito-chondria	0.48	−1.04	Involved in hypoxia-induced cell death of neuronal cells
Q16555	18/13	0.01	Dihydropyrimidinase-related protein 2 (*DPYSL2*)	Cytoplasm	0.48	−1.04	Plays a role in neuronal development and polarity, as well as in axon growth and guidance, neuronal growth cone collapse and cell migration
P04179	9/8	0.00	Superoxide dismutase [Mn], (*SOD2*)	Mito-chondria	0.48	−1.05	Destroys superoxide anion radicals
O96000	7/7	0.03	NADH dehydrogenase [ubiquinone] 1 beta subcomplex subunit 10 (*NDUFB10*)	Mito-chondria	0.48	−1.05	Accessory subunit of the mitochondrial membrane respiratory chain NADH dehydrogenase (Complex I)
P51649	12/12	0.03	Succinate-semialdehyde dehydrogenase, (*ALDH5A1*)	Mito-chondria	0.48	−1.05	Catalyzes one step in the degradation of the inhibitory neurotransmitter gamma-aminobutyric acid (GABA)
P23458	1/1	0.05	Tyrosine-protein kinase JAK1 (*JAK1*)	Cytoplasm	0.48	−1.06	Tyrosine kinase of the non-receptor type, involved in the IFN-alpha/beta/gamma signal pathway
P06732	69/41	0.03	Creatine kinase M-type (*CKM*)	Cytoplasm	0.48	−1.07	Reversibly catalyzes the transfer of phosphate between ATP and various phosphogens
O95139	3/3	0.04	NADH dehydrogenase [ubiquinone] 1 beta subcomplex subunit 6 (*NDUFB6*)	Mito-chondria	048	−1.07	Accessory subunit of the mitochondrial membrane respiratory chain NADH dehydrogenase (Complex I)
P40925	18/18	0.02	Malate dehydrogenase, cytoplasmic (*MDH1*)	Cytoplasm	0.47	−1.08	Malic enzyme activit
A8MU46	9/7	0.04	Smoothelin-like protein 1 (*SMTNL1*)	Nucleus	0.47	−1.09	Regulation of contractile properties of muscles
O95169	4/4	0.03	NADH dehydrogenase [ubiquinone] 1 beta subcomplex subunit 8, (*NDUFB8*)	Mito-chondria	0.46	−1.10	Accessory subunit of the mitochondrial membrane respiratory chain NADH dehydrogenase (Complex I)
P36957	14/12	0.03	Dihydrolipoyllysine-residue succinyltransferase component of 2-oxoglutarate dehydrogenase complex, (*DLST*)	Mito-chondria	0.46	−1.11	Dihydrolipoamide succinyltransferase (E2) component of the 2-oxoglutarate dehydrogenase complex
Q9NVS9	2/2	0.01	Pyridoxine-5′-phosphate oxidase (*PNPO*)	Cytoplasm	0.46	−1.12	Catalyzes the oxidation of either pyridoxine 5′-phosphate (PNP) or pyridoxamine 5′-phosphate (PMP) into pyridoxal 5′-phosphate (PLP)
Q9NPJ3	3/3	0.00	Acyl-coenzyme A thioesterase 13 (*ACOT13*)	Cytoplasm	0.46	−1.12	Catalyzes the hydrolysis of acyl-CoAs to the free fatty acid and coenzyme A
Q9P0J0	7/7	0.05	NADH dehydrogenase [ubiquinone] 1 alpha subcomplex subunit 13 (*NDUFA13*)	Mito-chondria	0.46	−1.13	Accessory subunit of the mitochondrial membrane respiratory chain NADH dehydrogenase (Complex I)
P51970	8/8	0.04	NADH dehydrogenase [ubiquinone] 1 alpha subcomplex subunit 8 (*NDUFA8*)	Mito-chondria	0.46	−1.13	Accessory subunit of the mitochondrial membrane respiratory chain NADH dehydrogenase (Complex I)
P00395	4/4	0.05	Cytochrome c oxidase subunit 1 (*MT-CO1*)	Mito-chondria	0.45	−1.17	Component of the respiratory chain that catalyzes the reduction of oxygen to water
P61019	4/4	0.00	Ras-related protein Rab-2A (*RAB2A*)	Endoplas-mic Reticulum	0.44	−1.19	Protein transport from the endoplasmic reticulum to the Golgi complex
O00217	3/3	0.04	NADH dehydrogenase [ubiquinone] iron-sulfur protein 8, (*NDUFS8*)	Mito-chondria	0.43	−1.20	Core subunit of the mitochondrial membrane respiratory chain NADH dehydrogenase (Complex I)
Q9UKU7	4/4	0.05	Isobutyryl-CoA dehydrogenase, (*ACAD8*)	Mito-chondria	0.43	−1.21	Plays a role in transcriptional coactivation within the ARC complex
P13798	8/8	0.00	Acylamino-acid-releasing enzyme (*APEH*)	Cytoplasm	0.43	−1.21	Catalyzes the hydrolysis of the N-terminal peptide bond of an N-acetylated peptide to generate an N-acetylated amino acid
P53778	3/2	0.05	Mitogen-activated protein kinase 12 (*MAPK12*)	Cytoplasm	0.42	−1.23	Essential component of the MAP kinase signal transduction pathway; In skeletal muscle colocalizes with SNTA1 at the neuromuscular junction and throughout the sarcolemma
O43676	1/1	0.03	NADH dehydrogenase [ubiquinone] 1 beta subcomplex subunit 3 (*NDUFB3*)	Mito-chondria	0.41	−1.26	Accessory subunit of the mitochondrial membrane respiratory chain NADH dehydrogenase (Complex I)
Q9NUU6	1/1	0.02	Inactive ubiquitin thioesterase FAM105A (*FAM105A*)	Information not available	0.40	−1.29	Information not available
Q93100	4/3	0.05	Phosphorylase b kinase regulatory subunit beta (*PHKB*)	Plasma membrane	0.36	−1.45	Catalyzes the phosphorylation of serine in certain substrates, including troponin I
Q8TAT6	3/3	0.01	Nuclear protein localization protein 4 homolog (*NPLOC4*)	Endoplas-mic Reticulum	0.36	−1.45	Binds ubiquitinated proteins and is necessary for the export of misfolded proteins from the ER to the cytoplasm, where they are degraded by the proteasome
Q9H7C9	4/4	0.02	Mth938 domain-containing protein (*AAMDC*)	Cytoplasm	0.36	−1.47	Plays a role in preadipocyte differentiation and adipogenesis
Q8N8N7	5/5	0.02	Prostaglandin reductase 2 (*PTGR2*)	Cytoplasm	0.29	−1.81	Functions as 15-oxo-prostaglandin 13-reductase; overexpression represses transcriptional activity of PPARG and inhibits adipocyte differentiation
P02792	4/4	0.01	Ferritin light chain (*FTL*)	Cytoplasm	0.27	−1.86	Stores iron in a soluble, non-toxic, readily available form
Q14558	1/1	0.01	Phosphoribosyl pyrophosphate synthase-associated protein 1 (*PRPSAP1*)	Cytoplasm	0.17	−2.57	Negative regulatory role in 5-phosphoribose 1-diphosphate synthesis
P29350	2/2	0.04	Tyrosine-protein phosphatase non-receptor type 6 (*PTPN6*)	Nucleus	0.15	−2.73	Modulates signaling by tyrosine phosphorylated cell surface receptors such as KIT and the EGF receptor/EGFR
Q8NDH3	1/1	0.00	Probable aminopeptidase NPEPL1 (*NPEPL1*)	Nucleus	0.13	−2.90	Catalyzes the removal of unsubstituted N-terminal amino acids from various peptides
P24043	50/48	0.00	Laminin subunit alpha-2 (*LAMA2*)	ECM	0.07	−3.84	Binding to cells via a high affinity receptor, laminin; mediates the attachment, migration, and organization of cells into tissues during embryonic development by interacting with other extracellular matrix components

*Pep, peptides; uniq, unique. Information concerning subcellular localization and protein function were obtained from uniprot. ECM, extra cellular matrix*.

Perturbed proteins represent tissue markers for *LAMA2*-related congenital myopathy and—based on information available in uniport—mainly localize to the sarcoplasm, to nuclei, the SR and to mitochondria thus suggesting a global unified organelle vulnerability in LAMA2-mutant skeletal muscle with a predominance to mitochondria. In addition, functions of several of the accord proteins accord with the known involvement of laminin-211 in ECM-composition, signal transduction, cytoskeleton, mitochondrial homeostasis and muscle cell development. Interestingly, functions of a variety of proteins increased in abundance hint toward the activation of neuroprotective mechanisms including neuromuscular transmission.

### Altered Abundance of Paradigmatic Proteins and Mitochondrial Vulnerability Can Be Confirmed in Independent Muscle Biopsies Derived From MDC1A-Patients

**Figure 4:**
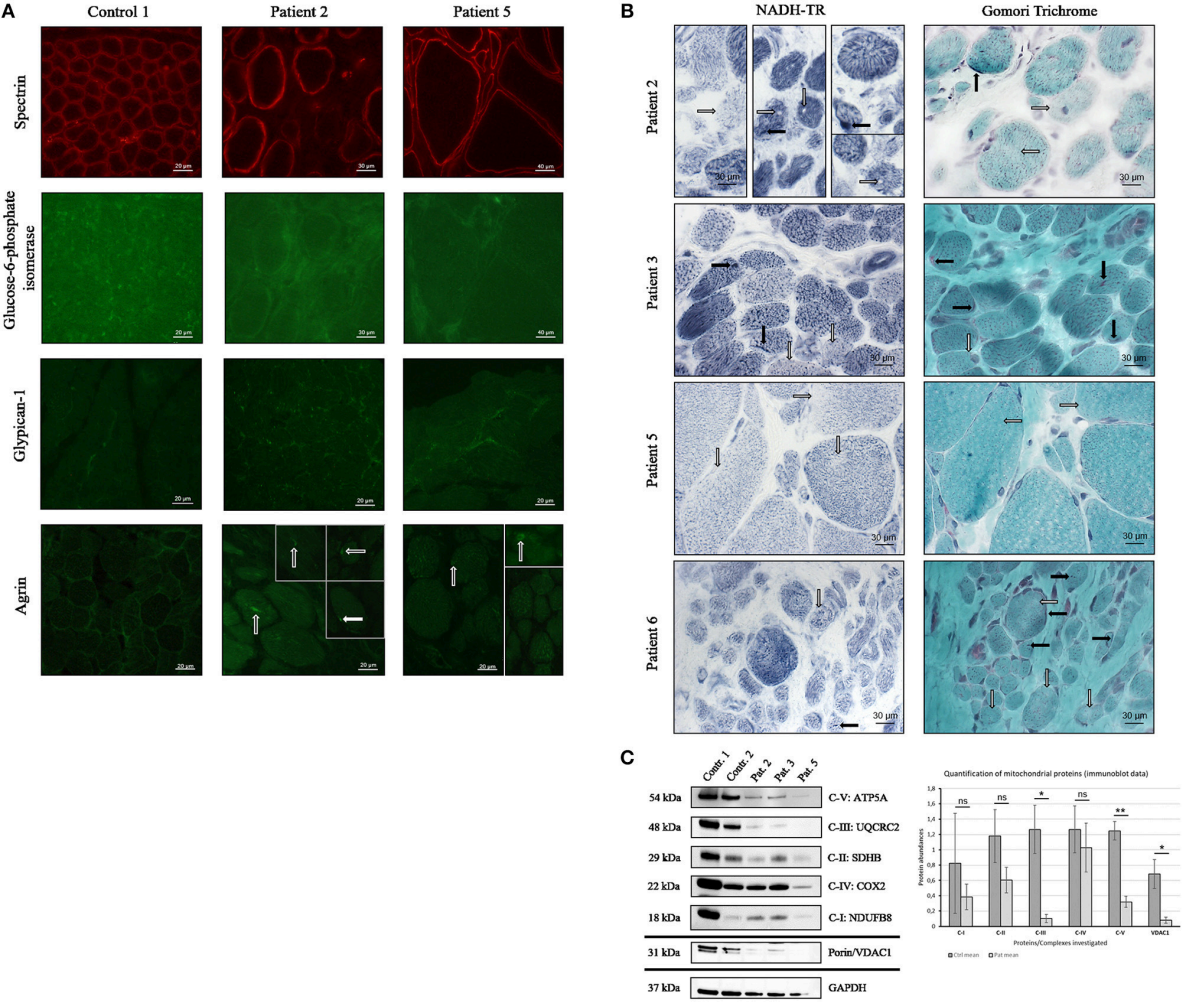
Confirmation study of proteomic findings in independent MDC1A-patient derived muscle biopsies. **(A)** Immunofluorescence studies of spectrin have been carried out to visualize sarcolemma in muscle cryostat sections of control and patient-derived muscles and to show similar abundance and localization of the protein. Further immunofluorescence studies of glucose-6-phosphate isomerase reveal a focal dot-like sarcoplasmic staining/ enrichment in muscle biopsy specimen derived of controls (one representative control is shown here) but show a decrease in the biopsies of two representative MDC1A-patients. Immunofluorescence studies of glypican-1 showed focal enrichment at the sarcolemma of control muscle (one representative control is shown). However, in the LAMA2-diseased muscle fibers, small dots sarcoplasmic, and (sub-)sarcolemmal dots immunoreactive for glypican-1 were detected. Immunofluorescence studies of agrin revealed of a sarcoplasmic enrichment/ aggregation in MDC1A-patient derived muscle fibers (white arrows) compared to control muscle fibers. **(B)** Histological NADH-TR staining revealed “sarcoplasmic gaps” (white arrows in patient 2; second and third column) and reduced enzyme activity (white arrows in patient 2, 3, 5, and 6) as well as focal enrichment in some myofibers (black arrows in patient 2, 3, and 6). Modified Gomori Trichrome staining revealed sarcoplasmic areas with minor staining (white arrows in patient 2, 3, 5, and 6) as well as areas of sarcoplasmic and subsarcolemmal enrichment (black arrows in patient 2,3, and 5). **(C)** Immunoblot analysis of proteins representative for the respiratory chain complexes I-V and of VDAC1 (porin) revealed a statistically significant decrease of complexes III and V as well as of VDAC1 in MDC1A-patient derived protein muscle extracts compared to controls (diagram). GAPDH has been used as loading control and proteins have been quantified against GAPDH (ns, not significant; *statistically significant, **statistically very significant).

To study the potential of altered proteins as tissue markers and reliability of our proteomic findings further immunofluorescence studies were performed on the muscle biopsies derived from the four patients included in the proteomic profiling (patients 1–4) as well as on biopsies derived from two further MDC1A-cases (patients 5 and 6). Doing so, we focused on abundances and distribution of agrin, glypican-1, and glucose-6-phosphate isomerase as paradigmatic proteins. Immunofluorescence-based studies of agrin showed occasionally focal sarcoplasmic accumulations most likely leading to the detected increase of overall agrin protein level (Table 3) as shown for two representative cases in Figure 4A. In comparison to the controls which show an enrichment of glypican-1 at the sarcolemma, in MDC1A-patient derived biopsies, small sarcoplasmic dots immunoreactive for the protein as well as an enrichment of these dots at the (sub-)sarcolemmal region could be observed. This irregular protein-distribution is shown for two representative cases in Figure 4A and most likely causes the 3.25-fold (log2 ratio) increase detected by proteomic profiling. Moreover, immunofluorescence studies confirmed the decreased expression of glucose-6-phosphate isomerase in muscle biopsies derived from four MDC1A-cases (shown for two representative cases in Figure 4A).

Given that our proteomic data revealed a profound vulnerability of NADH dehydrogenases, NADH muscle histology (NADH-TR to highlight the oxidative enzyme activity) has been investigated in biopsies of six MDC1A-cases and revealed no structural abnormalities like cores, whorles or lobulated fibers but reduced staining and even gaps in some muscle fibers indicative for reduced enzyme activity (shown for four representative cases in Figure 4B). Modified Gomori Trichrome staining has additionally been performed in these patients to study mitochondrial abnormalities and occasionally revealed both, focal accumulations and reduced staining suggesting irregular mitochondrial distribution (shown for four representative cases in Figure 4B). Immunofluorescence studies of Glucose-6-phosphate isomerase (GPI), glypican-1 and agrin have been carried out twice with similar results. Prompted by the proteomic results suggestive for vulnerability of the respiratory chain, respective complexes have been investigated by immunoblotting utilizing antibodies targeting proteins localized to the respective complexes and muscle protein extracts derived from two controls and three MDC1A-patients. Results of these studies confirm a vulnerability of the respiratory chain complexes (Figure 4C) and thus support our proteomic findings which were indicative for mitochondrial vulnerability. Immunoblot studies have been carried out twice with similar results.

## Discussion

A complete loss of laminin-211, which is encoded by *LAMA2*, causes a severe congenital muscular dystrophy with onset of symptoms in the first few months of life. Partial laminin-211 deficiency can be caused not only by primarily *LAMA2* mutations, but also secondarily by other muscular dystrophies, including dystroglycanopathy (26, 27). This in turn suggests the need to define tissue marker proteins for patients with classical MDC1A based on *LAMA2* mutations leading to laminin-211 deficiency. To systematically address this need, proteomic profiling has been carried out using muscle biopsy specimens derived from MDC1A patients with myopathy and brain malformations. Results not only confirm laminin-211 deficiency but also accord with elevated CK level in the patients as intramuscular CK was significantly decreased in the patient-derived muscle. In addition, decrease of Mth938 domain-containing protein (involved in preadipocyte differentiation and adipogenesis) as well as of prostaglandin reductase 2 (involved in inhibition of adipocyte differentiation) might provide a molecular link to a replacement of degenerating muscle fibers by fatty tissue. Based on the considerable increase of proteins localized to the extracellular matrix (Table 3) our data moreover confirm proliferation of connective tissue as a pathophysiological hallmark of fibrosis observed in the muscle biopsy specimen of our patients (Figure 1). The increase of LAMA4 (laminin subunit α4/ Lm-411) as well as of LAMA5 (laminin subunit α5/ Lm-511; identified by our routine diagnostic staining (Figure 2B) and 1.7-fold (log2 ratio) increased with a p-Anova of 0.19 in our proteomic findings; data not shown) most likely reflects a cellular attempt to (partially) compensate the loss of laminin-211 and to avoid a complete breakdown of muscle fibers. This assumption is not only supported by the results of a previous study showing that laminin-111 protein therapy reduces muscle pathology and improves viability of a MDC1A mouse model (28) but also by the known important role of LAMA4 for NMJ-integrity (29), the concomitant increase of integrin alpha-6/beta-1, a receptor for laminin and the increase of the basal cell adhesion molecule/ laminin alpha-5 receptor (Table 3) to optimize laminin binding to laminin-211-deficient muscle fibers. However, despite the increase of LAMA4 in MDC1A, the resultant laminin-protein complex is known to bind merely poorly to integrins and α-dystroglycan (30).

Given that subtle neuromuscular junction (NMJ) defects have been reported in laminin α2 chain-deficient mice (31), the detected abnormal sarcoplasmic accumulation and thus decreased release to the synaptic cleft of muscle agrin, a basal lamina glycoprotein crucial for the formation and the maintenance of NMJs, might contribute to impaired neuromuscular transmission as part of the MDC1A-pathophysiology. Along this line, increase of glypican-1, might indicate activation of a compensatory mechanism to avoid NMJ-breakdown resulting from profound de-innervation. Notably, mini-agrin has been shown to bind to the basement membrane and the DGC via α-dystroglycan and thus ameliorate muscle pathology *in vivo* (32) and transgenic expression of mini-agrin (contains binding sites for laminins and α-dystroglycan) and αLNNd (recombinant protein linking LAMA4 to α-dystroglycan) in a mouse model for MDC1A fully restored basement membrane stability. This effect resulted in recovery of muscle force and size leading to increased overall body weight, and extended life span (33). Moreover, glypican-1 has recently been linked to the pathophysiology of a muscular dystrophy complicated by a myasthenic syndrome (34) as well as of laminin α4, which has an important role in NMJ-integrity (29). However, the conclusion of endogenous activation of compensatory mechanisms in muscle of MDC1A-patients is further supported by the increase of C4b-binding protein alpha chain belonging to the complement system that deposits its activation products on innervated motor end-plates in ALS-patients (35).These combined findings in turn underline the significance our proteomic profiling data aiding the identification of marker proteins with pathophysiological relevance as well as impacts for attempts to develop new treatment strategies for MDC1A.

However, as the NMJ represents a paradigmatic synapse, altered abundance of a variety of other proteins including 14-3-3F, AKR7A2, protein FAM162A, GTPC1, NIBAN, PRVA, SRC8, Succinate-semialdehyde dehydrogenase, TTHY (Table 3), might correlate with the broad activation of neuroprotective mechanisms in the etiopathology of MDC1A and thus also link to the CNS alterations of the children. NudC domain-containing protein 2 modulates the LIS1/dynein pathway via stabilization of lissencephaly protein 1 (LIS1) with Hsp90, a cellular chaperone. Importantly, the p.L279P of NudC domain-containing protein 2 influences LIS1 stability (36). Given that lissencephaly has been described in MDC1A-patients (37) increase of this protein not only accords with the concept of activation of proteins involved in neuroprotection (in none of our patients lissencephaly was found via MRI) but also provides a first molecular hint to the manifestation of lissencephaly in the etiopathology of MDC1A. Neuronal vulnerability associated with altered synaptic transmission—as one pathophysiological cascade among others—is indicated by the decrease of glucose-6-phosphate isomerase, acting as a neurotrophic factor for spinal and sensory neurons (38), of phosphatidylethanolamine-binding protein 1 involved in the function of the presynaptic cholinergic neurons of the central nervous system, of dihydropyrimidinase-related protein 2 involved in neuronal development and polarity and in axon growth and guidance (Table 3). Activation of protective mechanisms toward maintenance of muscle contraction is for instance indicated by increase of ADDG, CNN3 and myosin light chain 4 as well as MYL6B (Table 3). Increased abundance of sarcolemmal membrane-associated protein involved in myoblast fusion (Table 3) might hint to activation or muscle fiber regeneration.

Interestingly, the results of our proteomic profiling indicate a profound vulnerability of mitochondria in laminin-211-deficient muscle based on the downregulation of a variety of mitochondrial proteins (Table 3 and Figure 4C) and reduced mitochondrial respiration and ATP production in MDC1A-patient derived muscle cells has been reported as the result of changes in abundances of transcripts encoding for mitochondrial key players (39). Among the dysregulated expression of genes related to energy production in myotubes, Fontes-Oliveira and colleagues described reduced level of *NDUFA8* and decrease of NADH dehydrogenase [ubiquinone] 1 alpha subcomplex subunit 8 encoded by this transcript has been identified in our study (Table 3). Thus, our proteomic data confirm an impairment of the mitochondrial bioenergetic status on the protein level in human MDC1A muscle cells also in complex laminin-211-deficient muscle progressed in the etiopathology. Clincally, one of our patients (patient 5) presented with rhabdomylosis and loss of the ability to stand and walk with support after a febrile infection. One might speculate that this unusual disease course also hints toward severe metabolic impairment.

As our study is based on the utilization of material derived from patients with progressed status of the disease, it is not possible to clearly differentiate between primary pathophysiological events and molecular changes occurring as a phenomenon secondary to the primary cascades. However, intersection with data obtained from skeletal muscle derived from murine animal models with early (and unified) manifestation of the disease might harbor some limitations regrading general validity of data comparison but still allows to define candidate marker proteins of general significance involved in the initiate pathophysiology. De Oliveira and co-workers studied the proteomic signature of diaphragm and gastrocnemius muscle derived from *dy*^3*K*^*/dy*^3*K*^ mice (MDC1A mouse model). Out of the approximately 700 identified proteins, 113 and 101 respectively, were differentially expressed in the investigated tissues (40). Notably, there was no overlap between the proteins increased in the muscle of the mouse model and the patients although few proteins with elevated levels in murine laminin-211-deficient muscle are related to extracellular matrix composition and might hint to early stages of fibrosis. Interestingly, parvalbumin alpha has been identified as decreased in their study but as increased in our proteomic profiling of MDC1A-patient derived quadriceps muscle. As in skeletal muscle, parvalbumin is thought to be involved in relaxation after contraction, its increase might accord with the activation of compensatory mechanisms upon disease progression. However, on a general note, one might speculate that either the differences in the investigated muscles (diaphragm and gastrocnemius in mice and vastus in human) or the progressed disease in our patients compared to the young-aged animals (four weeks) might explain the missing overlap between increased proteins in mice and human. Nevertheless, when focussing on the decreased proteins, a variety of different ones are downregulated in both, human and mice thus representing promising tissue markers for MDC1A across species. These proteins include creatine kinase M-type, voltage-dependent anion-selective channel protein 1, glucose-6-phosphate isomerase, cytoplasmic malate dehydrogenase, cytochrome C oxidase subunit 5A, mitochondrial NADH-dehydrogenase [ubiquinone] flavoprotein 1 and dihydrolipollysine-residue succinyltransferase component of 2-oxoglutarate dehydrogenase complex as well as NADH dehydrogenase [ubiquinone] 1β subcomplex subunit 10 and 2-oxoglutarate dehydrogenase.

The intersected data is indeed indicative of defective metabolism—especially mitochondrial function—in laminin-211-deficient muscle cells of MDC1A patients and hence confirm the findings of Fontes-Oliveira and colleagues in mice (39). This is moreover underlined by the results of our histological NADH-TR and Gomori Trichome staining supporting the concept of impaired mitochondrial activity. Along this line, the findings of both studies support the hypothesis that skeletal muscle metabolism might be a promising pharmacological target to improve muscle function, energy efficiency and tissue maintenance of MDC1A (39). Notably, in a pre-clinical study utilizing Lama2-deficient mice, mitochondria have been proven to represent a promising therapeutic target (41).

## Conclusion

Analysis of the proteomic signature of proximal muscle derived from four children suffering from MDC1A allowed the identification of 86 proteins with altered abundance and potential pathophysiological impact. Focussing on molecular changes in skeletal muscle, a variety of affected proteins are linked to the vulnerability of the central nervous system, especially to altered synaptic transmission in the disease caused by MDC1A. Moreover, this suggests that therapeutic intervention targeting the synaptic dysfunction might represent a promising element of the overall concept in the treatment of MDC1A as already shown in mice treated with agrin. Although our patients do not present with lissencephaly, increased NudC domain-containing protein 2 might represent a potential protein modifier of the central nervous system phenotype of MDC1A. Proteomic signature of laminin-211-deficient muscle moreover indicated a profound mitochondrial vulnerability with predominant decrease of proteins belonging to complex 1. A comparison of our data with the proteomic signature of laminin-211-deficient distal muscle and diaphragm from mice allowed the identification of nine decreased proteins representing potential markers for MDC1A. However, more comprehensive studies utilizing skeletal muscle derived from MDC1A-patients (sub-cohorts at different stages of the disease) are needed to ultimately define vulnerable proteins as definite MDC1A tissue markers.

## Contribution to the Field Statement

MDC1A is the most common form of congenital muscular dystrophies. The disease is caused by mutations in the *LAMA2* gene and molecular studies of mouse muscle and human cultured muscle cells already allowed first insights into the underlying pathophysiology. However, the definition of (candidate) marker proteins in human skeletal muscle is still lacking. To address this gap of knowledge, we conducted a study to investigate the proteomic signature of laminin-211-deficient vastus muscle obtained from four MDC1A-patients. Results of our unbiased screening allowed the identification of potential tissue marker proteins which might also be involved in the pathophysiology of the disease.

## Ethics Statement

Ethics commitee of Duisburg-Essen University.

## Author Contributions

HK conceptualized and designed the study, drafted the first version of the manuscript, interpreted results, and was principally responsible for the final content. DH carried out the initial analysis and interpretation of results, contributed to the discussion. AR carried out the initial analysis, conceptualized, and designed the study, reviewed and revised the manuscript for important intellectual content. RH and MJ performed the analysis of mitochondrial proteins. US reviewed and revised the manuscript for important intellectual content.

### Conflict of Interest Statement

The authors declare that the research was conducted in the absence of any commercial or financial relationships that could be construed as a potential conflict of interest.
